# Curve-Based Infill Pattern Optimization for 3D Printed Polymeric Scaffolds for Trabecular Bone Applications

**DOI:** 10.3390/ma18174055

**Published:** 2025-08-29

**Authors:** Gisela Vega, Rubén Paz, Mario Monzón, Ricardo Donate, Andrew Gleadall

**Affiliations:** 1 Mechanical Engineering Department, Universidad de Las Palmas de Gran Canaria, Campus de Tafira Baja, 35017 Las Palmas, Spain; mario.monzon@ulpgc.es (M.M.); ricardo.donate@ulpgc.es (R.D.); 2 Wolfson School of Mechanical and Manufacturing Engineering, Loughborough University, Loughborough LE11 3TU, UK

**Keywords:** optimization, scaffold, tissue engineering, additive manufacturing, modeling, FEA

## Abstract

Additive manufacturing technology, specifically material extrusion, offers great potential for scaffold manufacturing in tissue engineering. This study presents a novel methodology for the design and optimization of 3D printed polymeric scaffolds to enhance cell viability, thereby promoting improved cell proliferation for tissue engineering applications. Different infill patterns, including gyroid, parallel sinusoidal, and symmetric sinusoidal, were evaluated to determine their impact on cell proliferation and tissue regeneration. To overcome the limitations of existing slicer software, a novel open-source software called FullControl GCode Designer was utilized, enabling the creation of customized infill patterns without restrictions. VOLCO software was employed to generate voxelized 3D models of the scaffolds, simulating the material extrusion process. Finite element analysis was conducted using Abaqus software to evaluate the mechanical properties of the different designs. Additionally, new scripts were developed to evaluate the interconnectivity and pore size of the voxelized models. A factorial design of experiments and a genetic algorithm (combined with Kriging metamodels) were applied to identify the optimal configuration based on optimization criteria (keeping the mechanical stiffness and pore size within the recommended values for trabecular bone and maximizing the surface and interconnectivity). Biological testing was conducted on polylactic acid scaffolds to preliminarily validate the effectiveness of the modeling and optimization methodologies in this regard. The results demonstrated the agreement between the optimization methodology and the biological test since the optimum in both cases was a symmetric sinusoidal pattern design with a configuration resulting in a structure with 53.08% porosity and an equivalent pore size of 584 µm. Therefore, this outcome validates the proposed methodologies, emphasizing the role of pore surface area and interconnectivity in supporting cell proliferation. Overall, this research contributes to the advancement of AM technology in tissue engineering and paves the way for further optimization studies in scaffold design.

## 1. Introduction

Additive manufacturing (AM) and, in particular, material extrusion AM (MEX, according to ISO 52900:2021), commonly known as fused deposition modeling (FDM), is one of the most promising technologies for scaffold manufacturing in the tissue engineering field [1,2,3] due to the possibility of obtaining porous structures from a solid 3D model and of printing a wide range of biocompatible and bioactive materials.

Several authors use slicer software to generate the G-code file for printing the porous structure from a solid part [4,5,6], defining the geometrical and manufacturing parameters. Among the geometrical ones, the infill pattern, layer height, or infill density can be defined; consequently, several configurations of scaffolds can be obtained from the same solid.

The main mechanical requirement of the scaffold is to have a similar Young’s modulus to the tissue where it will be implanted. In this work, the scaffolds are designed for generic trabecular bones. For this type of tissue, some authors define its stiffness in the range of 10–1500 MPa [7,8].

According to research about cell growth and tissue regeneration through scaffolds, pores’ surface, shape, size, and interconnectivity are relevant for scaffolding design [9,10,11]. Regarding pore size, it is established that pores greater than 300 µm and smaller than 1 mm are desired [9,12,13,14,15]. Additionally, it is affirmed that the optimum value for bone formation is 500 µm [15].

On the other hand, for optimizing cell growth, it has been remarked that bone regeneration linearly increases with curvature, preferably on concave surfaces [9,10,11,16,17]. On ordinary slicer programs, the gyroid is the only pattern that fulfills the curvature requirements. However, it presents some limitations for scaffold design in tissue engineering. Specifically, its porosity can only be modulated by altering the unit cell (UC) size, as the extrusion width and wall thickness are fixed by the printing process. This constraint introduces a trade-off: increasing the UC size enhances porosity but may result in pore dimensions that exceed the optimal range for bone tissue regeneration, while decreasing the UC size to meet pore size requirements can lead to reduced porosity and an excessively high equivalent Young’s modulus. These limitations restrict the adaptability of gyroid-based scaffolds when simultaneously targeting mechanical compliance and biological performance.

Some researchers have discussed the constraints of the available slicer software [18], and others have developed an algorithm to obtain the G-code for their specific applications [19,20,21]. In this particular field of application, the main limitations are the narrow range of infill patterns and the inability to modify their parameters. A novel and open-source software called FullControl GCode Designer [22] is used to overcome constraints. It was created to cover a vast range of printing requirements and allows for designing any pattern through mathematical equations without restrictions.

This is the first study to design customized sinusoidal patterns for 3D printed polymeric scaffolds and compare them with other available curve-based patterns, such as gyroids, and implement a voxel-based modeling and a genetic algorithm optimization methodology to obtain the best configuration regarding infill pattern, surface, pores size, and interconnectivity for maximizing cell growth and tissue regeneration. These patterns contribute to the formation of interconnected pores with favorable sizes for cell migration and nutrient diffusion, which are critical for tissue regeneration. The three custom lattices used were gyroid, parallel sinusoidal, and symmetric sinusoidal. The first can be obtained in commercial or open-source slicing programs, and the others must be manually created in FullControl Gcode Designer. All of them were modeled with VOLCO, a software that generates a voxelized 3D model which simulates the material extrusion [23]. Previous research selected it as the best solution for the modeling and subsequent Finite Element Analysis (FEA) simulation of small and complex geometries [24].

Furthermore, this work develops a methodology to optimize the models from FEA results, taking into account the design parameters, in order to achieve suitable Young’s modulus and pore sizes for bone applications and, at the same time, maximize the surface of the scaffolds and the interconnectivity of the pores. The optimal designs were 3D printed and subjected to a biological test to validate the optimization conclusions.

## 2. Materials and Methods

### 2.1. Materials

Polylactic acid (PLA), a bioplastic with suitable properties for tissue engineering applications, was employed for printing scaffolds. In particular, the Smartfil PLA (Smart Materials 3D Printing S.L., Alcalá la Real, Spain) was used, whose properties are shown in Table 1.

### 2.2. Modeling and Optimization Method

**Figure 1:**
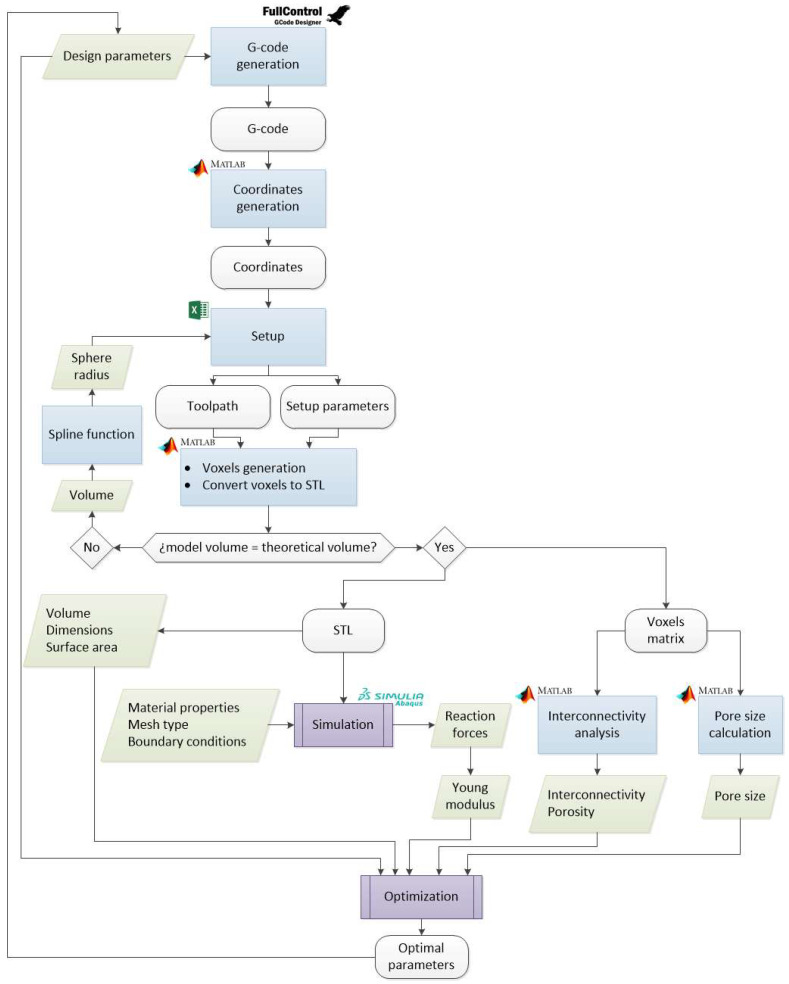
Methodology for modeling and optimizing sinusoidal scaffolds.

In order to optimize scaffolds for trabecular bone regeneration without printing or experimental testing, a modeling and optimization methodology with the application of FEA is required. The steps of the methodology followed are presented in Figure 1.

#### 2.2.1. Scaffold Design

**Figure 2:**
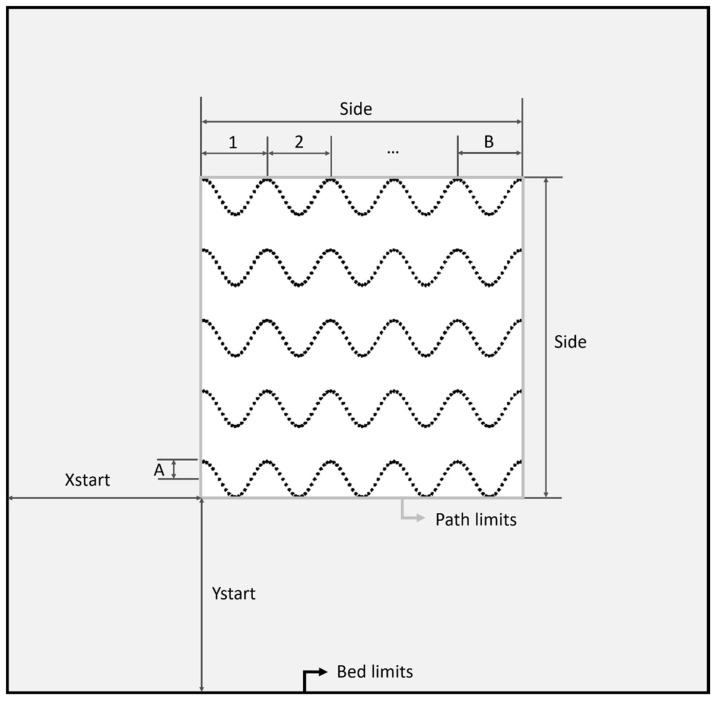
Design parameters for sinusoidal path generation in FullControl.

The main goal of the work is to obtain scaffolds with curvature to promote cell growth. For that aim, the slicing software Slic3r 1.3.0 was used. However, the only available curve pattern in commercial slicing software is gyroid. Therefore, another open-source software with fewer constraints, FullControl Gcode Designer 3.0, was used to design novel patterns for manufacturing scaffolds. The new pattern is based on sinusoidal curves and requires print settings and mathematical design to generate the paths, while the mathematical design needs parameters and equations to be defined. The parameters that represent the general geometry of the part (Figure 2) are part_side, whose value is the length and width of the square (in mm), and part_height, which is the height of the prism (mm). In this case, as a cubic scaffold was selected for the study, all these values were the same (part_side and part_height). To define the sinusoidal layout, A, B, and n_filaments represent the amplitude of the wave, its number of cycles, and the number of filament lines in each layer, respectively. Concretely, the number of filaments per layer will directly influence the porosity and distance between filaments. In addition, some printing parameters are required, such as Xstart and Ystart, which represent the XY coordinates of the printing bed (mm), where the part begins to be printed; layer_height, which is the height of each layer of filaments (mm); and fil_width, which is the width of the filament (mm).

The features used for modeling are the line equation to define the mathematical equation of the sinusoidal lines; the cartesian repeat to repeat identical sweeps (lines and layers); the line feature to define the travel movements of the nozzle; and the repeat rule to incrementally rotate layers 90°, interspersing horizontal and vertical paths. After including the necessary features and the mathematical equations to obtain a cubic scaffold with a sinusoidal pattern, the software generates the G-code file of the model.

To explore different curvature-based infill strategies, three sinusoidal patterns were defined: (1) a cosine equation repeated across filaments, (2) a sine equation with the same repetition logic, and (3) a combination pattern applying sine equations to odd layers and cosine equations to even layers. Unlike the first two patterns, where each layer is rotated 90°, the combined sine-cosine pattern applies the rotation every two layers.

**Figure 3:**
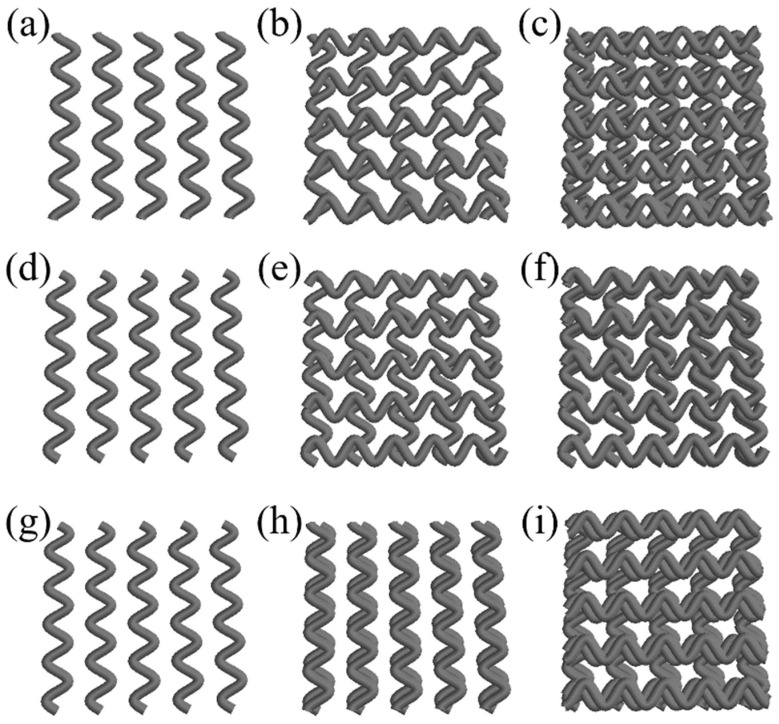
Differences among sinusoidal infill patterns. (**a**) First layer of cosine pattern. (**b**) Two layers of cosine pattern. (**c**) Four layers of cosine pattern. (**d**) First layer of sine pattern. (**e**) Two layers of sine pattern. (**f**) Four layers of sine pattern. (**g**) First layer of cosine and sine combination pattern. (**h**) Two layers of cosine-sine pattern. (**i**) Four layers of cosine-sine pattern.

Figure 3 illustrates representative layers of each pattern, serving as a visual reference to validate the correct implementation of the mathematical logic and rotation rules in the design process. These examples were generated to ensure that the intended geometrical variations were accurately reflected in the scaffold architecture prior to final modeling.

**Figure 4:**
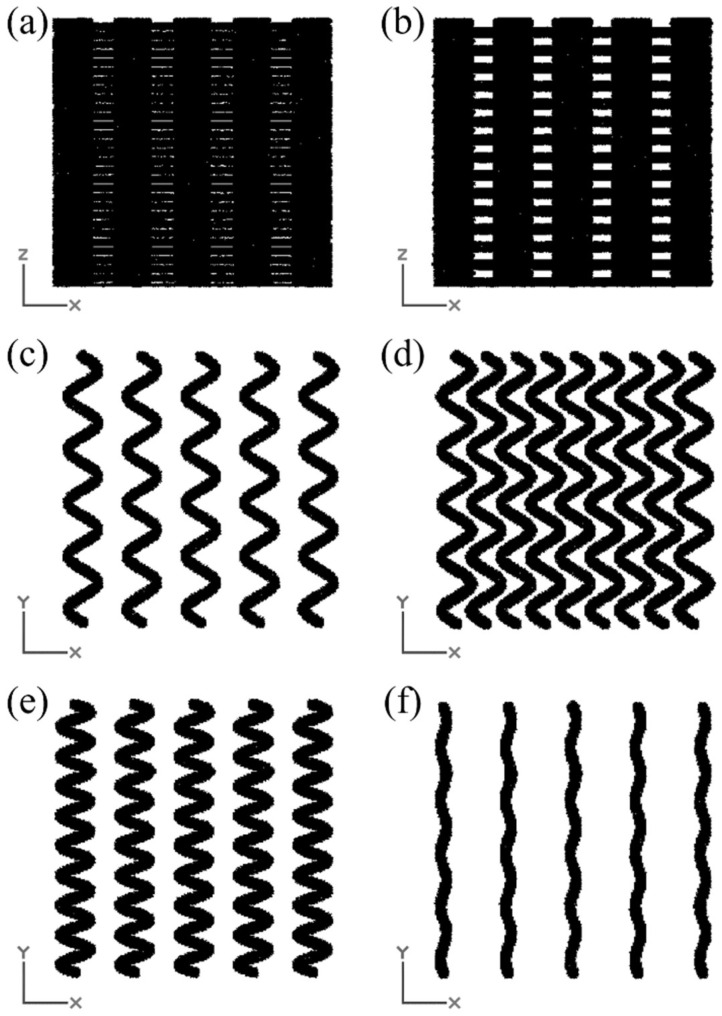
Geometric differences of 9 × 9 × 9 mm sinusoidal scaffolds depending on the values of variables. (**a**) Scaffold with a layer height of 0.15 mm (front view). (**b**) Scaffold with a layer height of 0.3 mm (front view). (**c**) Filament layout with an amplitude of 0.5 mm, 5 cycles, and 5 filaments per layer. (**d**) Filament layout with an amplitude of 0.5 mm, 5 cycles, and 9 filaments per layer. (**e**) Filament layout with an amplitude of 0.5 mm, 9 cycles, and 5 filaments per layer. (**f**) Filament layout with an amplitude of 0.1 mm, 5 cycles, and 5 filaments per layer.

Moreover, to illustrate the influence of design parameters on the scaffold geometry, a series of representative configurations was generated using FullControl Gcode Designer. Figure 4 presents examples of the resulting filament layouts for different values of layer height, amplitude, number of cycles, and number of filaments per layer. These visualizations were used to verify the correct implementation of the parametric design logic and to ensure that the generated geometries reflect the intended variations in scaffold architecture.

**Figure 5:**
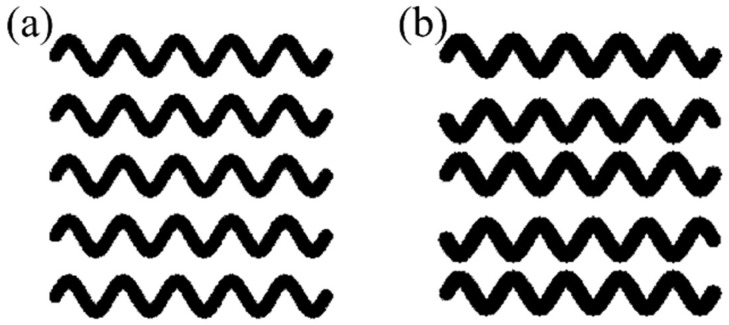
Difference between filament configurations. (**a**) Parallel filaments or sinusoidal. (**b**) Symmetric filaments or reflected sinusoidal.

After following the modeling and optimizing methodologies of sinusoidal scaffolds, the optimal pattern is introduced as the default pattern of a new reflected sinusoidal scaffold whose filaments are symmetric instead of parallel to determine which shape is better for cell growth. The difference between parallel and symmetric filaments is shown in Figure 5.

In the case of reflected sinusoidal scaffolds, the number of variables was reduced to four, compared with the five variables of the previous design, because the optimal infill pattern was used as the default one in this new configuration. Therefore, the number of samples designed was reduced to sixteen. The variables and their values are shown in Table 2.

In contrast with the preceding groups, gyroid scaffolds were designed through slicing software (Slic3r 1.3.0); thus, parameters and mathematical equations are not modifiable. Consequently, the two selectable variables are porosity, which modifies the filament layout, and layer height. The selected values result in four samples (Table 2).

In summary, the modeling and optimization methodologies are applied to three groups of scaffolds whose difference is their pattern: sinusoidal, reflected sinusoidal, and gyroid. The first two groups were designed with custom print-path software, and the variation between the sinusoidal pattern and the reflected one is the geometric characteristics of the filaments: parallel or symmetric, respectively.

#### 2.2.2. Modeling

The next step in the modeling process is obtaining a voxelized model in STL format from the G-code file. It is done with VOLCO software [23], which predicts the microarchitecture of the 3D geometry, simulating the material extrusion during the manufacturing process. The filaments are represented as voxelized spheres deposited one after the other, and when one interacts with any obstacle, it simulates the expansion of the material in the available space, keeping the volume constant.

To start generating the voxelized part, VOLCO requires the toolpath coordinates. Therefore, a coordinates generator script was designed in Matlab R2021a to obtain them from the G-code. Apart from coordinates, VOLCO software needs other parameters for voxel simulation and STL generation, such as the cuboid region for the 3D model, voxel size, layer height, or sphere radius.

In this work, an adjustment procedure was implemented to determine the appropriate sphere radius for each layer height during voxelization. For that aim, a representative filament line was modeled for each layer height, and an iterative refinement step was applied to improve the accuracy of the resulting voxelized volume. This step involves applying a cubic spline interpolation through the spline function of Matlab, which adjusts the sphere radius to ensure that the voxelized filament volume closely matches the theoretical volume defined in the G-code. This procedure was explicitly applied to the scaffolds designed with FullControl Gcode Designer.

In the case of the gyroid scaffolds, the spline function was iteratively applied to the volume to adjust the sphere radius of each configuration.

After defining the values of the parameters, the G-code was obtained by FullControl and voxel matrix and voxelized models in VOLCO. On the other hand, the G-code of the gyroid scaffolds was generated by Slic3r 1.3.0 and the voxel matrix and 3D model by VOLCO.

The 3D voxel model was used to determine pore size, equivalent Young’s modulus, interconnectivity, porosity, and surface area.

#### 2.2.3. Interconnectivity and Porosity

In scaffolding applications, porous structures are desired for cell growth, bringing an intrinsic porosity. The optimal value of this porosity is not set; it is considered a result of the structure. On the other hand, the interconnectivity of the pores is essential for fluid driving, cell migration, and tissue ingrowth. Therefore, it must be maximized.

Firstly, the method used for interconnectivity analysis [25] was based on the concept of the Effective Pore Connectivity Index (EPCI) [26]. It is the proportion of connected voxels from the last layer to the first.

Nevertheless, to overcome the limitations of EPCI, the authors decided to apply the 6-connected neighborhoods algorithm using the “bwconncomp” Matlab function [25]. Other researchers have used a similar approach [27,28]. Some authors consider interconnectivity as a fraction of the volume of voids accessible from outside and the total volume of the structure [27]. On the other hand, others additionally constrain narrow connections [28].

In contrast with previous studies that calculate interconnectivity from a 3D binary matrix generated from Computer Tomography images, this work begins from the voxels matrix obtained in VOLCO. The first step is to reverse the matrix and set the zero value to material and the one to void voxels. Then, the interconnectivity of the pores is defined by the Connected Volume Index (CVI) value, particularly the two sides criteria, which is calculated considering the volume of the groups of voids that connect any two external faces using Equation (1).

(1) CVI(%)=Volume of groups of voids that link two external faces/Total volume 

Finally, the porosity is calculated in the same subroutine by dividing the volume of voids and the total volume, as it is not a value already defined in the design process.

#### 2.2.4. Pore Size

The size of the pores is relevant for cell growth. It is not desirable to have either too small or too large pores. Considering this premise and the bibliography, it was determined that the acceptable range of pore sizes would be between 300 and 700 µm [9,12,13,14,15].

**Figure 6:**
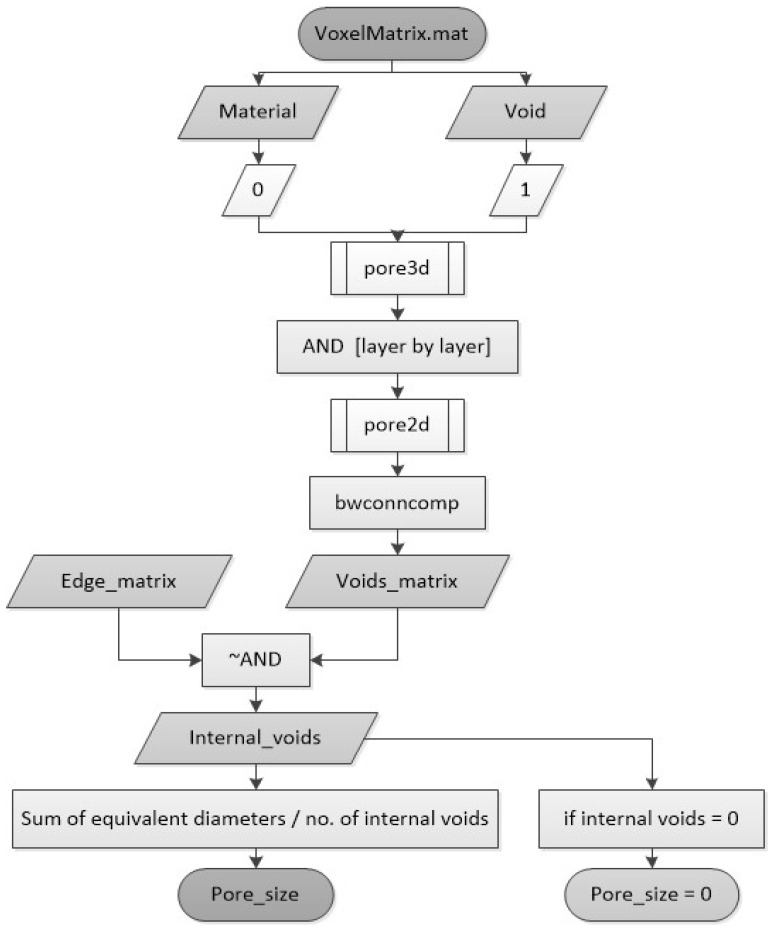
Pore size calculation process.

**Figure 7:**
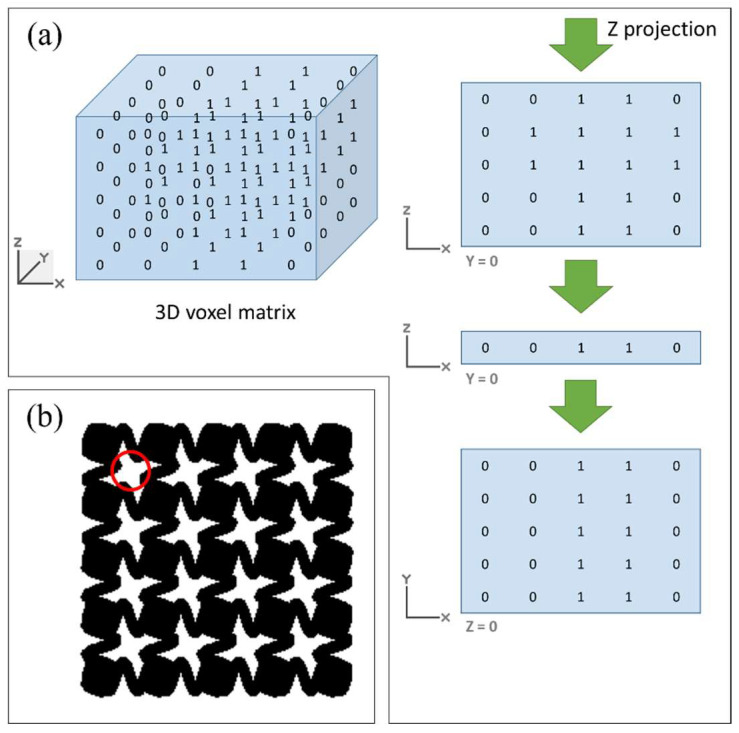
Pore size determination. (**a**) 3D voxel matrix and its Z projection. (**b**) Equivalent pore diameter (red circle).

To evaluate whether the scaffold designs meet the pore size requirements for tissue engineering applications, an automated tool was developed to calculate the equivalent pore diameter. The process is summarized in the diagram shown in Figure 6. It starts from the same 3D matrix used for interconnectivity analysis, which defines whether there is a void or material in each voxel. Then, a Boolean operation (AND) is used layer-by-layer in the Z coordinate to obtain the Z projection in a 2D matrix. Figure 7a shows an example of the matrix projection.

Once the Z projection is obtained in a 2D matrix, the pores of this matrix are identified with the “bwconncomp” function [25]. Then, they are analyzed to determine if they are adjacent to the edges (external pores) or not (internal pores). The pore size will be calculated only with the internal pores. The equivalent diameter of each pore is determined, and then all the results are summed and divided by the number of internal pores, resulting in the average equivalent diameter of all holes, thus the pore size. Figure 7 illustrates this methodology, showing an example of the Z-projection of a voxelized scaffold and the identification of internal pores used for diameter calculation. This figure serves as a visual reference to clarify the computational steps involved in the pore size analysis and to ensure reproducibility of the method.

#### 2.2.5. Surface

Another objective to be maximized in scaffold structures is the surface area, which is associated with the potential area available for cell deposition. However, the actual distribution or attachment of cells on the scaffold surface was not directly assessed in this study.

This value can be extracted from the STL file of the model or directly in Abaqus/CAE 6.14-1 before meshing the part (analyzing the mass properties with the query tool).

#### 2.2.6. Finite Element Analysis

After obtaining the model from the G-code file by VOLCO, the 3D model in STL format is imported into Abaqus/CAE 6.14-1 to apply the FEA.

The first step is to convert the triangular elements into tetrahedrons, obtaining the meshed model. The mesh was composed of linear tetrahedral elements (C3D4 type). The mesh was generated automatically from the voxel-based geometry, with each element corresponding to a voxel size of 50 μm.

Then, the material properties of PLA are assigned to the entire scaffold, using a Young’s modulus of 3861 MPa and a Poisson’s ratio of 0.35, based on the manufacturer’s data. Next, a new step (Step-1) is created to establish it as the final step of the simulation. After that, the boundary conditions are defined to simulate a compression test. For that aim, the Z displacement is prevented on the bottom face of the part (base set); also, two points of the base, placed side by side, are encastred to avoid rotation without preventing lateral expansion during compression, and a Z displacement of 0.2 mm is applied on the top face of the part (to emulate the compression).

The significant simulation results are the displacement (U) and reaction forces (RF) obtained in the deformed scaffold. The equivalent Young’s modulus is calculated from the reaction forces obtained in the bottom face (base set) in the Z direction, using Equation (2), extracted from ISO 604:2002 (plastics—determination of compressive properties).

(2) $$E=\sigma /\varepsilon =F\cdot L_{0}/(A\cdot ∆L)$$

where E is the equivalent Young’s modulus or modulus of elasticity (MPa), σ is the tensile stress (MPa), ε is the deformation, F is the reaction force in the Z direction (N), A is the equivalent area (mm^2^), ΔL is the displacement (mm), and L_0_ is the original height (mm). Note that the equivalent area corresponds to the area of the base as if it were a solid part. Consequently, the equivalent modulus of elasticity represents the general mechanical stiffness of the scaffold, including the effect of the pores.

#### 2.2.7. Optimization

**Figure 8:**
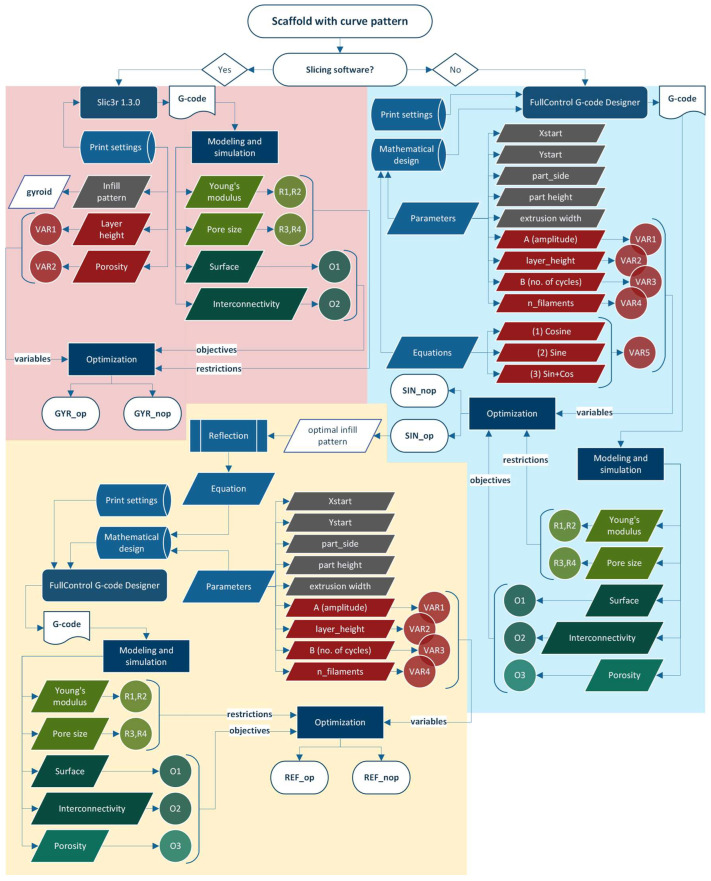
Optimization diagram of scaffolds with curve-based infill patterns: parallel sinusoidal scaffold (blue), symmetric sinusoidal scaffold (yellow), and gyroid (red).

The optimization was done with an evolutionary algorithm, specifically a genetic algorithm. In order to start with the genetic algorithm optimization process, all the variables, objectives, and constraints must be previously obtained or calculated. A summary of the whole process is shown in Figure 8.

**Figure 9:**
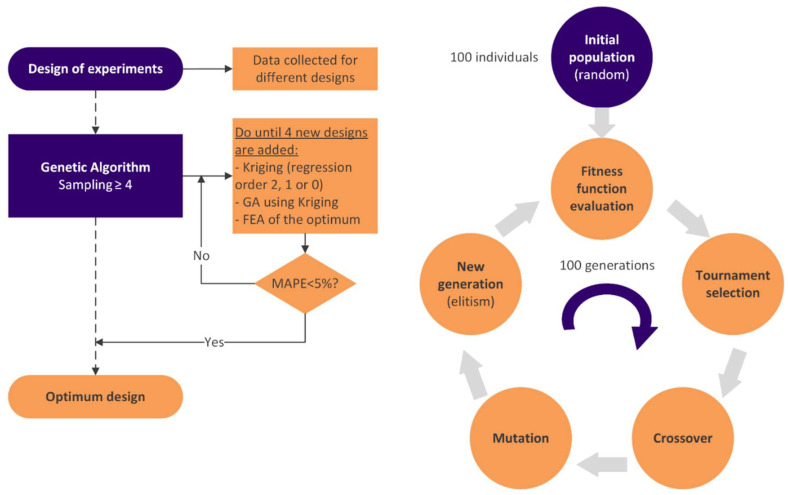
Optimization process.

Regarding the optimization step, it uses a tournament selection of two individuals, an arithmetic crossover, mutation, reparation, and elitism after calculating the fitness function for each individual (Figure 9). The genetic algorithm evolutes for 100 generations, each with 100 individuals. The initial population is randomly generated, and then the algorithm assesses the fitness function for each sample. Each response was evaluated using the Kriging metamodel interpolation method [29,30].

The information obtained throughout the earlier factorial design of experiments served as the basis for the metamodels. The Kriging metamodel employs an exponential correlation model and a polynomial regression model, with the distinction that the order of the polynomial regression model varies based on the data from order 2 up to 0. The development of the metamodel is made simpler using a lower order, but accuracy decreases. Therefore, better estimates can be obtained using the 2-order regression model, but this requires more or more evenly spread samples. Due to this, the optimization algorithm has a loop that first employs a 2-order regression model to predict each response and immediately switches to a 1-order regression model if the development of the metamodel is unsuccessful (and so on for the 0-order). As a result, the optimal regression model based on the available data is always employed [30,31].

The genetic algorithm generates a random number between 0 and 1 for the crossover and each design variable. If the value is less than or equal to 0.5 (50% crossover probability), the first offspring will inherit the value of the first parent, and the second offspring will inherit the value of the second parent. If not, the first child will inherit the second parent’s value, and the second child will inherit the first parent’s value. Thus, genetic information is exchanged between the parents, resulting in two offspring with mixed characteristics.

After the crossover, a random value is produced for each individual in the resulting population (between 0 and 1). The associated individual is mutated if this value is lower than or equal to 0.6 (60% mutation probability). The mutation entails picking a random design variable and adding the outcome of a random value (between −0.5 and 0.5) multiplied by the maximum interval of that design variable to the existing one to alter it slightly. A round operation is used to get an integer value for the discrete variables. In the event that the mutation causes the new designs to fall outside the defined search space, a repair is performed on these variables, establishing the limit value. Thus, a new generation is created, and elitism is applied to preserve the best individual of the previous generation.

In this case, five variables, two constraints, and three objectives are considered for optimization. Depending on the application, the infill pattern; the porosity; and the A, B, n_filaments, and layer_height design parameters can be defined as variables.

In the case of constraints, it must be selected whether it is a maximum or a minimum. Nevertheless, in this study, restrictions are defined as an interval; thus, a minimum and a maximum must be set for each. Consequently, each restriction must be split into two; model compression modulus results are used in constraints R1 and R2, and pore size in R3 and R4. Concretely, the equivalent elastic modulus must be greater than 10 MPa (R1) and smaller than 1500 MPa (R2), and the pore size must be larger than 300 µm (R3) and smaller than 700 µm (R4).

Moreover, the shared objectives are the surface area (O1) and interconnectivity (O2), but porosity (O3) is an objective in the case of sinusoidal scaffolds (parallel and symmetric). This last objective is not included in the fitness function; it is only obtained to know its value.

The fitness function is presented in Equation (3) and maximizes the objective result, which considers the objectives O1 and O2 equally weighted at 50%, normalizing their values around their average values. Moreover, if the optimization restrictions are not met during the process, a penalty factor reduces the fitness value.

(3) $$\mathrm{Fitness}\;\mathrm{function}=\max\;(0.5\cdot O1/\overset{-}{O1}+0.5\cdot O2/\overset{-}{O2})$$

The genetic algorithm is applied to the initial set of samples to predict a theoretical optimal configuration using the Kriging metamodel. If the proposed configuration is already included among the samples from the design of experiments, it is considered valid, and the optimization process is concluded. Otherwise, the new design must be modeled and simulated using the previously established methodology, and the results must be added to the population as additional sampling data. This process is repeated iteratively until four new configurations not present in the initial dataset have been incorporated into the optimization loop.

Finally, establishing the new design as optimal must fulfill the mean absolute percentage error (MAPE) condition. As shown in Equation (4), MAPE is calculated and must be less than 5% to validate the new design.

(4) $$\mathrm{MAPE}=(100\%\;/\;n)\;\sum_{t=1}^{n}|(A_{t}-F_{t})/A_{t}|\;<\;5\%$$

where A_t_ represents the actual value of the constraints and objective results, F_t_ represents the forecasted values, and n is the number of outcomes compared.

In summary, Table 2 enumerates the variables, restrictions, and objectives for the optimization process. In particular, equivalent Young’s modulus and pore size are optimization constraints, with a minimum and a maximum value; thus, each one is considered twice. Regarding objectives, surface area and interconnectivity need to be maximized. Nevertheless, in the scaffold obtained by FullControl Gcode Designer, porosity is also an objective because its value is unknown.

### 2.3. Biological Test

#### 2.3.1. Cell Culture and Seeding

Human bone marrow mesenchymal stem cells (hBMSCs) (SCC034, Merck KGaA, Darmstadt, Germany) were cultured in a low serum formulation medium (Human Mesenchymal-LS Expansion Medium, SCM023, Merck KGaA) supplemented with 1% of an antibiotic-antimycotic solution (A5955-100ML, Merck KGaA). The cells were cultured in 75 cm^2^ flasks (T75, Sarstedt AG & Co. KG, Nümbrecht, Germany) previously coated with a 0.1% gelatin solution (ES-006-B, EmbryoMax, Merck KGaA). For cell detachment, Trypsin-EDTA in Hank’s balanced salt solution (0.25% Trypsin and 1 mM EDTA, without Ca^2+^ and Mg^2+^, SM-2003-C, Merck KGaA) was used at 80–90% confluence.

The scaffolds to be seeded were 3.5 mm in height and had a 9 × 9 mm square base. They were manufactured by FDM in an Ender-3 3D printer (Shenzhen Creality 3D Technology Co., Ltd., Shenzhen, China) with a bed temperature of 60 °C and a nozzle temperature of 210 °C. Furthermore, the extrusion width is defined as 0.48 mm, and the filament diameter is 1.75 mm. Four replicas were used per group: SIN_op, GYR_op, REF_op, SIN_nop, GYR_nop, REF_nop.

The scaffolds were immersed in a 75% *v*/*v* ethanol solution, placed in a non-treated 24-well plate (Thermo Scientific™ Nunc™, Thermo Fisher Scientific, Suzhou, China), and later exposed to UV light under the fume hood for 1 h. Then, they were washed twice with a culture medium in order to remove any trace of ethanol. After placing the samples in a new well plate, the scaffolds were kept in an incubator (at 37 °C and 5% CO_2_) immersed in 1 mL of fresh culture medium for 1 h. After this period of sample hydration, the culture medium of each sample was replaced with a 1 mL suspension containing 30,000 hBMSCs. The culture medium was changed after the first 24 h.

#### 2.3.2. Cell Metabolic Activity Evaluation

Cell metabolic activity was evaluated using the CCK-8 protocol (Cell Counting Kit-8, Dojindo Molecular Technologies, Inc., Kumamoto, Japan). After 4 days of cell culture, the scaffolds were transferred to a new non-treated 24-well plate, and 0.7 mL of a 10% *v*/*v* solution of the CCK-8 reagent in culture medium was added. The same solution was poured into 4 empty wells that were used as negative controls. The plate was incubated at 37 °C and 5% CO_2_ for 3.5 h. Then, two 100 µL aliquots were transferred from each well to a 96-well reader plate (Thermo Scientific™ Nunc™—MicroWell™, Thermo Fisher Scientific). A BioTek ELx800 reader (Bio Tek Instruments Inc., Winooski, VT, USA) was used to measure the absorbance of the samples at an excitation wavelength of 450 nm. The result from the negative control group was subtracted from the absorbance obtained for each sample group tested.

### 2.4. Statistical Analysis

Statistical analysis was performed using MATLAB software (MATLAB and Statistics Toolbox Release 2021a, The MathWorks, Inc., Natick, MA, USA). The Kruskal–Wallis test was used for data analysis. The significance level was set to * *p* < 0.05, ** *p* < 0.01, and *** *p* < 0.001 for statistically significant, highly statistically significant, and very highly statistically significant differences, respectively. Figures showing statistical results show the mean values of each group and their standard deviations represented with error bars.

## 3. Results and Discussion

### 3.1. Preliminary Optimization

This work modeled and optimized three groups of scaffolds: sinusoidal, reflected sinusoidal, and gyroid. However, it was required to previously optimize the sinusoidal scaffold in order to obtain the optimal infill pattern, which was used as the default in the reflected sinusoidal one. After introducing the forty-eight samples (Table 3) into the genetic algorithm, the fifth sample is found to be optimal. It is a scaffold with five filaments per layer and a layer height of 0.15 mm. It is worth noting that the mathematical equation of the filaments corresponds to a sine equation, with five cycles and an amplitude of 0.5 mm.

### 3.2. Modeling and Simulation Results

As mentioned before, in the modeling process to obtain the sinusoidal and reflected sinusoidal scaffolds, it is required to previously apply an iterative function to a filament to determine the size of the sphere radius for each layer height. From the iteration results, sphere radii were selected: 134 µm for scaffolds with a layer height of 0.15 mm and 188.2 µm for those with a layer height of 0.3 mm. Then, they were defined as default in VOLCO.

On the other hand, the spline function was also applied to the whole gyroid scaffolds to adjust their sphere radius and, thus, their volume. In particular, the sphere radii of each sample, according to their printing parameters (layer height and infill percentage), are 199.565 µm (0.3 mm and 50%), 199.18 µm (0.3 mm and 60%), 199.24 µm (0.15 mm and 50%), and 199.17 µm (0.15 mm and 60%).

After the modeling and simulation processes, the results of the equivalent elastic modulus (R1, R2); pore size (R3, R4); surface area (O1); interconnectivity (O2); and, in the case of those scaffolds obtained by FullControl, porosity (O3) are showed in Table 3, Table 4 and Table 5.

In the case of sinusoidal samples from 19 to 24, they presented simulation errors, so compression modulus results had to be predicted with the metamodel created for the rest of the results, making an interpolation.

### 3.3. Optimization Process

**Figure 10:**
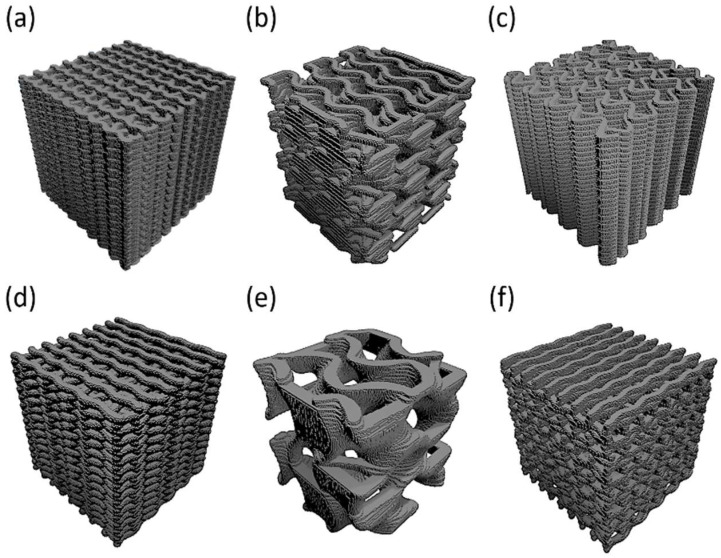
Optimal and non-optimal scaffolds geometry. (**a**) REF_op. (**b**) GYR_op. (**c**) SIN_op. (**d**) REF_nop. (**e**) GYR_nop. (**f**) SIN_nop.

**Figure 11:**
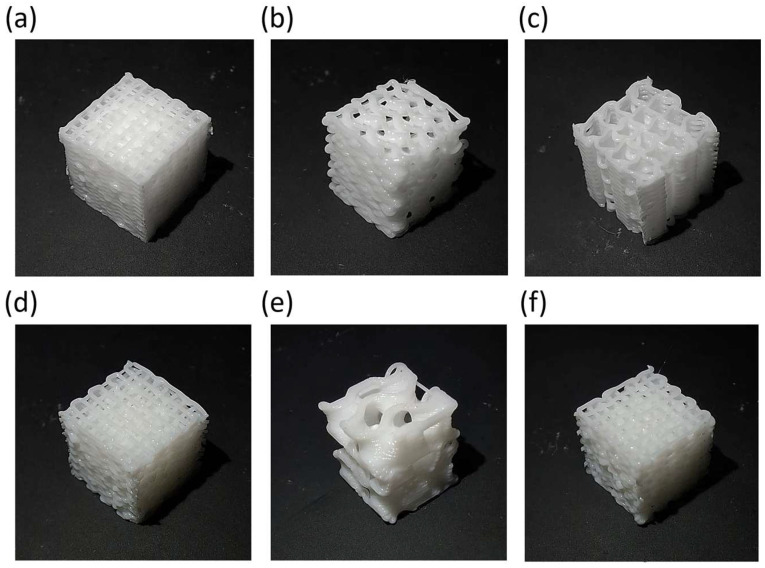
Optimal and non-optimal 3D printed scaffolds. (**a**) REF_op. (**b**) GYR_op. (**c**) SIN_op. (**d**) REF_nop. (**e**) GYR_nop. (**f**) SIN_nop.

The genetic algorithm results, the optimal combination for each configuration, were already modeled and simulated in all cases (combinations already analyzed in the design of experiments). Therefore, four iterations and MAPE verification were not required. Moreover, in order to biologically compare the performance of the samples and verify the correct working of the optimization process, the worst combination among the ones that fulfilled the restrictions for each case was also determined. In agreement with the fitness function, optimal and non-optimal configurations are presented in Table 6, the models’ geometrical results are shown in Figure 10, and 3D printed scaffolds are presented in Figure 11.

**Figure 12:**
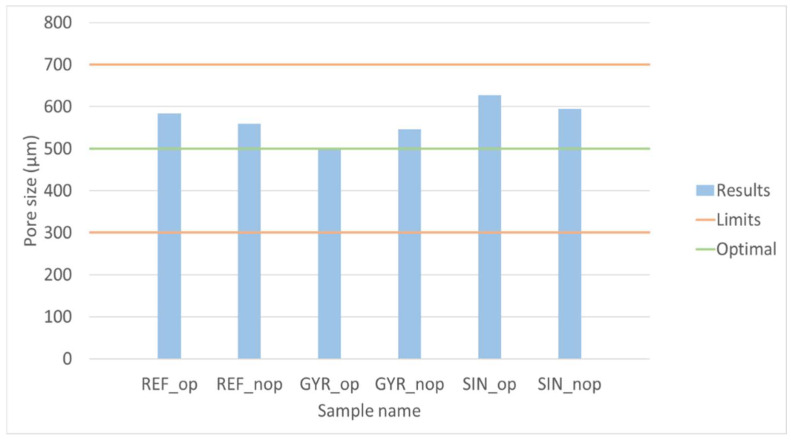
Models pore size compared with the optimal value.

**Figure 13:**
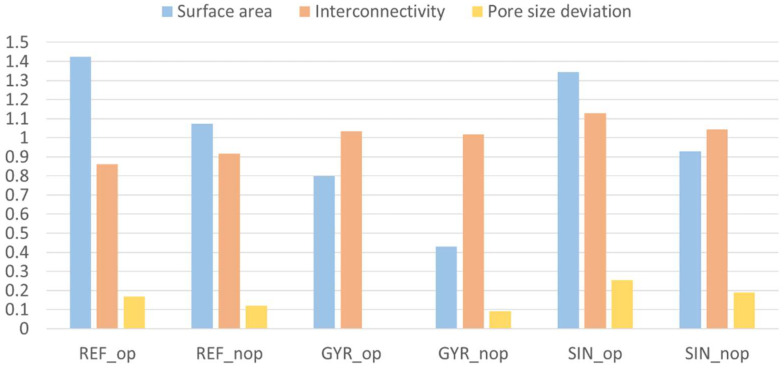
Comparison of the pondered values of the objectives, ordered according to the weighted average value.

The comparison of the six samples was studied in order to predict which would be optimal considering the different layouts. To make the objectives comparable, they were normalized, dividing their values by the average value of all samples. In addition, as mentioned before, the pore size in trabecular bones must be between 300 and 700 µm, according to Collins et al. [15]; it was 500 µm. Therefore, the influence of the deviation of the pore size results from the hypothetical optimum was studied. In Figure 12, the deviation of the models’ pore size from the optimal value is shown. The comparison of the normalized objectives (surface area and interconnectivity) and the normalized deviation of the pore size are shown in Figure 13.

Several comparison criteria were used, varying the weight of each objective (surface area and interconnectivity) in determining the optimal model for cell proliferation. The weights applied were 50/50 (Equation (5)), 40/60 (Equation (6)), and 60/40% (Equation (7)). These specific weightings were selected to explore the influence of biological versus mechanical priorities in the optimization process.

(5) $$\mathrm{fitness}\;\mathrm{function}\;(50/50\%)=0.5\cdot O1/\overset{-}{O1}+0.5\cdot O2/\overset{-}{O2}$$

(6) $$\mathrm{fitness}\;\mathrm{function}\;(40/60\%)=0.4\cdot O1/\overset{-}{O1}+0.6\cdot O2/\overset{-}{O2}$$

(7) $$\mathrm{fitness}\;\mathrm{function}\;(60/40\%)=0.6\cdot O1/\overset{-}{O1}+0.4\cdot O2/\overset{-}{O2}$$

**Figure 14:**
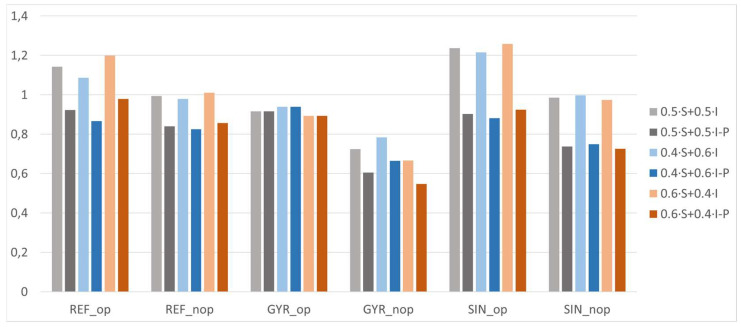
Comparison criteria for optimal model determination. S = surface area, I = interconnectivity, P = penalty.

The 50/50 weighting was initially chosen to equally prioritize both surface area and interconnectivity, which are widely recognized as critical parameters for promoting cell adhesion, proliferation, and nutrient diffusion. The alternative weightings (40/60 and 60/40) were introduced to assess the sensitivity of the optimization outcome to shifts in emphasis between these two objectives. Additionally, a penalty factor was applied based on the deviation from the biologically optimal pore size (*PS*), defined as 500 µm by the literature, to ensure that the optimization remained biologically relevant; in particular, the value of this penalty (P) is the third part of the absolute deviation after applying a unity-based normalization (Equation (8)). The results of each weighted average are shown in Figure 14.

(8) $$P=1/3\;\cdot \left(abs\left(PS-500\right)\;-{\;PS}_{min}\right)/\left({PS}_{max}\;-{\;PS}_{min}\right)$$

The ordered models from the most to the least optimal according to each criterion are shown in Table 7. The conclusions that can be extracted from the results are that the hypothetical optimal configurations are generally at the top of the ranking as long as the penalty is applied. The penalty favors the GYR_op model because its pore size is very close to 500 µm, and it is considered the best configuration when more weight is given to interconnectivity and the penalty is applied (40/60% with penalty criteria). When there is no penalty, SIN_op is considered the optimal one. REF_op is the best configuration if the objectives are weighted 50/50 or the surface area is more relevant (60/40%), applying the penalty in all cases. Note that the fitness function applied in the optimization process of each lattice was equivalent to that of 50/50. However, the average value used in the normalization of the objectives was different for each layout. For that reason, if the six models are compared in terms of the fitness function of the genetic algorithm and the 50/50 criterion, the ranking is not exactly the same.

Although the genetic algorithm did not generate new configurations beyond those defined in the design of experiments, its application was crucial to identify the most suitable scaffold designs systematically. The GA enabled the evaluation of multiple objectives and constraints simultaneously, providing a robust method for selecting optimal configurations that might not be evident through direct inspection of the DOE results alone. This highlights the value of incorporating evolutionary algorithms in scaffold design optimization, even when working within a predefined sample space.

In this context, it is generally observed that optimal designs tend to be located at the vertices of the search space. Therefore, a 2^n^ factorial design of experiments was applied to ensure that these boundary configurations were evaluated. Under these conditions, it is reasonable that the optimal solution coincides with one of the sampled points.

### 3.4. Biological Test Results

**Figure 15:**
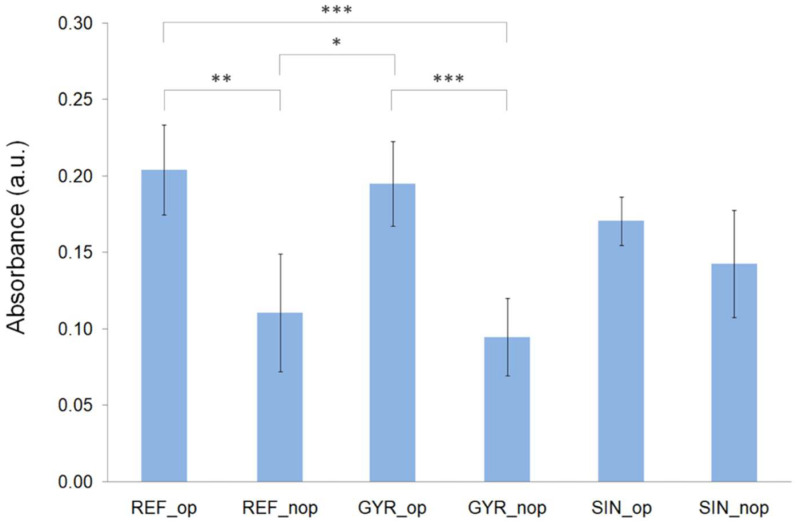
Metabolic activity of human bone marrow mesenchymal stem cells cultured on the different groups of scaffolds determined by the CCK-8 assay at day 5 (* *p* < 0.05, ** *p* < 0.01, and *** *p* < 0.001).

According to the results of the CCK-8 assay (Figure 15), after 5 days of culture, cells were viable in all groups tested. The highest mean values of absorbance, and therefore the highest metabolic activities of hBMSCs cultured on the scaffolds, were observed for the GYR_op and REF_op groups. Both groups showed statistically significant differences concerning their non-optimized counterparts, the GYR_nop and REF_nop groups. Additionally, a very highly statistically significant difference (*** *p* < 0.001) was obtained between the REF_op and GYR_nop scaffolds, which showed the highest and lowest mean absorbance values, respectively.

It should be noted that the CCK-8 assay measures the formazan produced by intracellular dehydrogenases, which is proportional to the number of metabolically active cells. Consequently, the observed signal may reflect not only differences in the number of cells attached and proliferating on the scaffolds but also variations in the metabolic activity of individual cells. This limitation should be considered when interpreting the influence of porosity on cell response.

The optimization methodology, including the factorial design of experiments and the genetic algorithm, has determined the best parameters’ values to obtain the optimal configuration of every pattern. On the other hand, as the hypothetical optimal models have outperformed the non-optimal ones, it can be considered that pore size influences biological behavior. Among the three comparison criteria with penalty studied, the one that better predicts the optimal configuration according to the experimental results, among all the patterns, is 50/50%. Only the fourth and fifth positions differ; thus, it could be used for the primary purpose of finding the optimal configuration for cell proliferation.

Analyzing the final result, the optimal configuration must be the reflected sine wave, with an amplitude of 0.1 mm, 9 cycles, and 9 filaments in each layer of 0.15 mm height. The equivalent elastic modulus of the model is 1175.36 MPa, which fulfills the requirements of trabecular bones, being between 10 and 1500 MPa; the pore size is 584 μm (between 300 and 700 μm and near the optimal of 500 μm); the surface area where cells can be deposited is 4822.78 mm^2^; and the interconnectivity of pores and porosity values is 53.08%.

**Figure 16:**
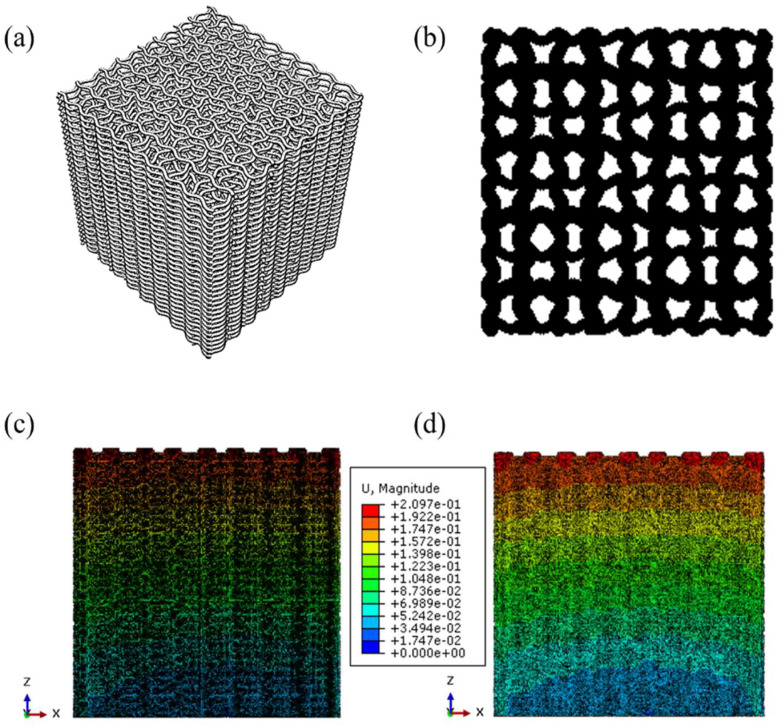
Optimal sample configuration. (**a**) Geometry. (**b**) Z-projection. (**c**) Non-deformed scaffold in the XZ plane. (**d**) Displacement of the deformed scaffold in the XZ plane.

The geometry, Z projection, and simulation results of the optimal configuration are shown in Figure 16.

### 3.5. New Criteria Application

In order to validate the 50/50% with penalty optimization criteria, it was applied to all groups. The optimal configurations for the gyroid and symmetric sinusoidal scaffolds are the same as those previously obtained. However, the GA has proposed a new optimal configuration for parallel sinusoidal scaffolds. Specifically, two new configurations were identified: specimen 49, with an amplitude of 0.5 mm, 5 cycles, 6 filaments per layer, a layer height of 0.15 mm, and a sinusoidal infill pattern, and specimen 50, which is similar but with a slightly reduced amplitude of 0.48 mm.

New biological tests must be performed to validate the new results. Nevertheless, the influence of the geometry, whether the filaments are symmetric or parallel, should be analyzed, and its integration into the optimization process must be studied. The main difference between the layouts is the shape of the channels where the cells grow. In the parallel filaments, the channels in each layer shape a curved path, and in the symmetric configuration, they form rings.

## 4. Conclusions

After applying a preliminary biological test to PLA cubic scaffolds, the viability of the modeling and optimization methodologies was demonstrated to identify the most suitable configuration, in terms of geometrical parameters, to support cell adhesion and metabolic activity. Although tissue regeneration was not directly assessed, the results provide a promising foundation for future in-depth biological evaluations.

The voxelized models of the scaffolds were generated in VOLCO, and the FEA was performed in Abaqus 6.14-1. The factorial design of experiments and genetic algorithm application (combined with Kriging metamodels) have contributed to predicting the best configuration for regenerating trabecular bone tissue according to the selected optimization criteria (keeping the mechanical stiffness and pore size within the recommended values for trabecular bone and maximizing the surface and interconnectivity). The seventh sample of the symmetric sinusoidal scaffold was the optimal design (according to the optimization methodology and the biological tests), representing 4822.78 m^2^ of surface area, 53% of interconnectivity, and an equivalent pore size of 584 µm. This pattern had to be designed in FullControl GCode Designer because it is unavailable in slicing software.

Therefore, the modeling and optimization methodologies proposed in this work are suitable and significantly advantageous in tissue engineering applications, such as porous structures, as demonstrated in this case for bone regeneration. Moreover, it was proven that the available surface and interconnectivity of the pores are relevant in the study of cell proliferation and tissue regeneration. Nevertheless, the influence of the filaments’ layout, with a symmetric and parallel distinction, in terms of cell proliferation should be studied and added to the optimization process.

Future work will include experimental validation of the mechanical and porosity properties of the printed scaffolds. In particular, mechanical validation will be addressed in greater depth, considering that this study focused on a generic trabecular bone model with a wide Young’s modulus range (10–1500 MPa), where simulation results are considered acceptable but may differ from experimental values in more constrained scenarios.

Further biological tests are required to gain a more complete understanding of how design influences the biological response. Such studies would involve long-term evaluations of proliferation, differentiation, and mineralization, together with fluorescence and FESEM imaging to assess cell growth and distribution. These aspects, however, fall beyond the scope of the present work, which mainly focuses on the design stage and incorporates biological testing only as a means of validating the optimization outcomes.

In conclusion, this research significantly contributes to AM technology’s progress in tissue engineering and lays the foundation for further optimization studies in scaffold design.

## Figures and Tables

**Table 1:** Smartfil PLA properties.

Material Properties of PLA—Smartfil	
Diameter	1.75 mm
Density	1.24 g/cm^3^
Tensile Strength	114 MPa
Elongation at Break	100%
Tensile Modulus	3861 MPa
Elmendorf Tear	13 g/mL
Print Temperature	220 ± 20 °C
Hot Pad	0–60 °C
Heat Deflection Temperature	65 °C
Vicat Softening Temperature	85 °C

**Table 2:** Design of experiments.

Group	Variables		Values	Restrictions		Objectives	
Sinusoidal	VAR1	Amplitude (mm)	0.1, 0.5	R1,R2	Young’s modulus	O1	Surface area
	VAR2	layer_height (mm)	0.15, 0.3			O2	Interconnectivity
	VAR3	B (cycles)	5, 9	R3,R4	Pore size	O3	Porosity *
	VAR4	n_filaments	5, 9				
	VAR5	Infill pattern	1 (cos), 2 (sin), 3 (cos + sin)				
Reflected sinusoidal	VAR1	Amplitude (mm)	0.1, 0.5	R1,R2	Young’s modulus	O1	Surface area
	VAR2	layer_height (mm)	0.15, 0.3			O2	Interconnectivity
	VAR3	B (cycles)	5, 9	R3,R4	Pore size	O3	Porosity *
	VAR4	n_filaments	5, 9				
Gyroid	VAR1	layer_height (mm)	0.15, 0.3	R1,R2	Young’s modulus	O1	Surface area
	VAR2	Porosity (%)	50, 70	R3,R4	Pore size	O2	Interconnectivity

* Porosity (O3) is reported only for designs developed using FullControl Gcode Designer and is not included in the fitness function.

**Table 3:** Results of sinusoidal scaffolds.

SAMPLE	VAR1 (mm)	VAR2 (mm)	VAR3	VAR4	VAR5	R1, R2 (MPa)	R3, R4 (µm)	O1 (mm^2^)	O2 (%)	O3 (%)
1	0.5	0.3	5	5	1	104.37	352.90	3227.82	70.58	70.59
2	0.5	0.3	5	5	2	262.71	635.61	3369	70.59	70.59
3	0.5	0.3	5	5	3	50.06	549.62	3262.06	70.59	70.59
4	0.5	0.15	5	5	1	63.63	351.72	4289.07	69.69	69.70
5	0.5	0.15	5	5	2	226.87	627.40	4550.87	69.69	69.70
6	0.5	0.15	5	5	3	11.59	143.11	4342.28	69.69	69.70
7	0.5	0.3	5	9	1	919.59	133.02	5098.52	47.04	47.06
8	0.5	0.3	5	9	2	1241.58	231.03	5118.21	47.05	47.06
9	0.5	0.3	5	9	3	648.44	0.00	5382.06	47.05	47.06
10	0.5	0.15	5	9	1	649.85	156.46	6073.25	45.44	45.47
11	0.5	0.15	5	9	2	1182.57	245.09	6023.88	45.44	45.47
12	0.5	0.15	5	9	3	30.33	59.18	6697.14	45.40	45.46
13	0.5	0.3	9	5	1	516.05	242.82	4012.04	56.83	56.85
14	0.5	0.3	9	5	2	760.54	1241.40	4070.59	56.60	56.62
15	0.5	0.3	9	5	3	1076.63	793.13	3703.97	56.84	56.85
16	0.5	0.15	9	5	1	361.19	307.99	5235.3	55.46	55.47
17	0.5	0.15	9	5	2	652.57	255.49	5415.32	55.70	55.71
18	0.5	0.15	9	5	3	270.14	912.24	4562.62	55.69	55.71
19	0.5	0.3	9	9	1	564.31 *	56.42	4609.76	22.16	22.33
20	0.5	0.3	9	9	2	644.57 *	0.00	4914.08	21.58	21.91
21	0.5	0.3	9	9	3	610.35 *	0.00	4546.22	21.36	22.33
22	0.5	0.15	9	9	1	496.53 *	83.50	5321.86	19.75	19.85
23	0.5	0.15	9	9	2	619.24 *	58.61	6048.98	20.07	20.28
24	0.5	0.15	9	9	3	409.47 *	0.00	5032.75	20.08	20.28
25	0.1	0.3	5	9	1	446.87	606.92	3528.91	64.42	64.42
26	0.1	0.3	5	9	2	544.52	731.10	3527.88	64.42	64.42
27	0.1	0.3	5	9	3	551.10	594.93	3142.19	64.42	64.42
28	0.1	0.15	5	9	1	330.80	732.59	4540.49	63.44	63.44
29	0.1	0.15	5	9	2	517.13	827.24	4521.83	63.44	63.44
30	0.1	0.15	5	9	3	410.48	659.61	3735.44	63.44	63.44
31	0.1	0.3	9	9	1	535.30	610.42	3829.16	62.38	62.39
32	0.1	0.3	9	9	2	605.39	637.16	3841.97	62.38	62.39
33	0.1	0.3	9	9	3	606.23	564.31	3401.87	62.39	62.39
34	0.1	0.15	9	9	1	430.24	705.68	4865.84	61.33	61.33
35	0.1	0.15	9	9	2	573.01	748.56	4886.62	61.33	61.33
36	0.1	0.15	9	9	3	496.30	631.74	3999.08	61.33	61.33
37	0.1	0.3	5	5	1	114.34	1887.70	2121.09	80.24	80.24
38	0.1	0.3	5	5	2	165.76	2015.30	2118.5	80.23	80.24
39	0.1	0.3	5	5	3	148.50	1842.70	1829.11	81.16	81.17
40	0.1	0.15	5	5	1	88.24	1991.70	2855.25	79.69	79.69
41	0.1	0.15	5	5	2	155.94	2101.40	2847.43	79.69	79.69
42	0.1	0.15	5	5	3	112.98	1815.60	2268.07	81.07	81.07
43	0.1	0.3	9	5	1	147.83	1587.90	2315.75	79.10	79.10
44	0.1	0.3	9	5	2	182.56	1776.70	2318.08	79.10	79.10
45	0.1	0.3	9	5	3	176.06	1559.40	1987.34	80.11	80.12
46	0.1	0.15	9	5	1	119.39	1860.20	3110.26	78.52	78.52
47	0.1	0.15	9	5	2	172.49	1755.30	3119.82	78.52	78.52
48	0.1	0.15	9	5	3	141.86	1706.30	2453.67	80.01	80.01

* Results of equivalent Young’s modulus obtained by interpolation.

**Table 4:** Results of reflected sinusoidal scaffolds.

SAMPLE	VAR1 (mm)	VAR2 (mm)	VAR3	VAR4	R1, R2 (MPa)	R3, R4 (µm)	O1 (mm^2^)	O2 (%)	O3 (%)
1	0.1	0.15	5	5	384.75	1095.5	3063.87	75.61	75.61
2	0.1	0.3	5	5	99.84	1015.8	2313.63	75.87	75.87
3	0.1	0.15	5	9	1071.58	1015.8	4524.43	75.62	75.62
4	0.1	0.3	5	9	667.33	559.39	3631.86	56.56	56.56
5	0.1	0.15	9	5	405.02	925.04	3342.48	73.93	73.93
6	0.1	0.3	9	5	107.72	890.29	2515.22	74.49	74.49
7	0.1	0.15	9	9	1175.36	584.00	4822.78	53.08	53.08
8	0.1	0.3	9	9	745.06	465.47	3913.4	54.08	54.09
9	0.5	0.3	5	5	380.68	981.94	3368.68	64.12	64.13
10	0.5	0.15	5	9	2250.45	244.24	4029.16	35.18	35.25
11	0.5	0.3	5	9	1352.73	227.11	3920.02	36.08	36.10
12	0.5	0.15	9	5	1464.82	422.17	4607.59	46.78	46.87
13	0.5	0.3	9	5	968.80	269.26	3765.03	47.39	47.40
14	0.5	0.15	9	9	3413.70	0	861.47	12.65	12.67
15	0.5	0.3	9	9	3365.57	0	960.14	10.56	10.57
16	0.1	0.15	5	5	384.75	1095.5	3063.87	75.61	75.61

**Table 5:** Results of gyroid scaffolds.

SAMPLE	VAR1 (mm)	VAR2 (%)	R1, R2 (MPa)	R3, R4 (µm)	O1 (mm^2^)	O2 (%)
1	0.3	50	567.45	500.83	2703.41	63.77
2	0.3	70	239.37	408.31	1702.67	77.45
3	0.15	50	1234.88	732.89	2090.98	42.11
4	0.15	70	534.22	545.91	1460.05	62.82

**Table 6:** Optimal and non-optimal samples.

Configuration	Sample	Young’s Modulus (MPa)	Pore Size (µm)	Surface Area (mm^2^)	Interconnectivity (%)	Porosity (%)	Fitness Function	Name
Reflected	7	1175.36	584.00	4822.78	53.08	53.08	1.23	REF_op
Reflected	4	667.33	559.39	3631.86	56.56	56.56	1.08	REF_nop
Gyroid	1	567.45	500.83	2703.41	63.77	50.00	1.20	GYR_op
Gyroid	4	534.22	545.91	1460.05	62.82	70.00	0.88	GYR_nop
Sinusoidal	5	226.87	627.40	4550.87	69.69	69.70	1.15	SIN_op
Sinusoidal	27	551.10	594.93	3142.19	64.42	64.42	0.93	SIN_nop

**Table 7:** Ranking of models according to each comparison criterion.

Comparison Criteria		0.5·S + 0.5·I	0.5·S + 0.5·I-P	0.4·S + 0.6·I	0.4·S + 0.6·I-P	0.6·S + 0.4·I	0.6·S + 0.4·I-P
Ranking	1	SIN_op	REF_op	SIN_op	GYR_op	SIN_op	REF_op
	2	REF_op	GYR_op	REF_op	SIN_op	REF_op	SIN_op
	3	REF_nop	SIN_op	SIN_nop	REF_op	REF_nop	GYR_op
	4	SIN_nop	REF_nop	REF_nop	REF_nop	SIN_nop	REF_nop
	5	GYR_op	SIN_nop	GYR_op	SIN_nop	GYR_op	SIN_nop
	6	GYR_nop	GYR_nop	GYR_nop	GYR_nop	GYR_nop	GYR_nop

## Data Availability

The original contributions presented in this study are included in the article. Further inquiries can be directed to the corresponding authors.
